# Data on conceptual design of cryogenic energy storage system combined with liquefied natural gas regasification process

**DOI:** 10.1016/j.dib.2017.09.015

**Published:** 2017-09-14

**Authors:** Inkyu Lee, Jinwoo Park, Il Moon

**Affiliations:** Department of Chemical and Biomolecular Engineering, Yonsei University, Republic of Korea

## Abstract

This paper describes data of an integrated process, cryogenic energy storage system combined with liquefied natural gas (LNG) regasification process. The data in this paper is associated with the article entitled “Conceptual Design and Exergy Analysis of Combined Cryogenic Energy Storage and LNG Regasification Processes: Cold and Power Integration” (Lee et al., 2017) [1]. The data includes the sensitivity case study dataset of the air flow rate and the heat exchanging feasibility data by composite curves. The data is expected to be helpful to the cryogenic energy process development.

**Specifications Table**

**Table:** 

Subject area	Chemical Engineering
More specific subject area	Process Systems Engineering
Type of data	Figures and Tables
How data was acquired	Through the computational process simulation by software, Aspen HYSYS
Data format	Filtered and analyzed
Experimental factors	Air flow rate, heat exchanging feasibility
Experimental features	Feasibility check, sensitivity analysis, performance evaluation
Data source location	Department of Chemical and Biomolecular Engineering, Yonsei University, Republic of Korea
Data accessibility	Data with this article

**Value of the data**

• The increase of the air flow rate causes increase of the power generation.
• The increase of the air flow rate causes decrease of the minimum temperature difference of the heat exchanger.
• The pinch point can be shown not only at the inlet or outlet but also inside the heat exchanger when the phase changing occurs.

## 1 Data

In this data article, we share sensitivity analysis data of the cryogenic energy storage system combined with liquefied natural gas (LNG) regasification process. In this data, the case study simulation results by air flow rate and the heat exchanging composite curves are illustrated.

## 2 Experimental design, materials and methods

### 2.1 Sensitivity analysis of the air flow rate

The air is used as the working fluid in this integrated energy storage system. To find the optimal flow rate of the air, the simulation case study is performed by the air flow rate for the sensitivity analysis. The flow rate of the LNG is fixed as 1.00 kg/s for every case. We set five cases of the air flow rate as follows: Case 1 is 0.40 kg/s, Case 2 is 0.45 kg/s, Case 3 is 0.50 kg/s, Case 4 is 0.55 kg/s and Case 5 is 0.60 kg/s of air flow rate. The specific work output of the cryogenic energy release system by the air flow rate is shown in Table 1. The stream notations are shown in Fig. 2 in Ref. [1].

**Table 1:** Specific work output by the air flow rate (kJ/kg-LNG).

**Specific work output**	**Case 1**	**Case 2**	**Case 3**	**Case 4**	**Case 5**
Air expander 1	30.23	34.01	37.79	41.57	45.35
Air expander 2	31.98	35.97	39.97	43.97	47.97
Air expander 3	33.27	37.42	41.58	45.74	49.90
Air expander 4	33.26	37.42	41.58	45.74	49.89
Total work output	128.74	144.82	160.92	177.02	193.11

The simulation result shows that the total work output is almost linear to the air flow rate.

### 2.2 Feasibility analysis for the heat exchangers

The detailed heat exchanging simulations are performed to check the feasibility and the results are shown in Table 2. The pinch temperature is set as 3 °C for every heat exchanger as the constraint. Therefore, the minimum temperature difference of the heat exchanger have to be larger than 3 °C. Finding pinch point is an important part in the procedure of the heat exchanging feasibility check. The hot stream is air and the cold stream is LNG for all heat exchangers. The cold LNG is vaporized via heat exchange with air, from HX1 to HX5. On the other hand, the air flows into HX5, then passes HX4, HX3, HX2, and HX1 successively. The air is pressurized between the heat exchangers and liquefied by HX5. Thus, the phase changing is occur inside the HX5.

**Table 2:** Minimum temperature difference and pinch point.

		**hot inlet side Δ*T* (K)**	**hot outlet side Δ*T* (K)**	**Minimum Δ*T* (K)**	**Pinch point**
**Case 1**	HX1	86.1	12.5	12.5	Hot stream outlet
	HX2	63.8	15.1	15.1	Hot stream outlet
	HX3	120.5	15.9	15.9	Hot stream outlet
	HX4	108.4	9.5	9.5	Hot stream outlet
	HX5	97.3	5.2	5.2	Hot stream outlet
					
**Case 2**	HX1	74.3	12.5	12.5	Hot stream outlet
	HX2	85.8	12.8	12.8	Hot stream outlet
	HX3	106.4	14.1	14.1	Hot stream outlet
	HX4	95.2	7.8	7.8	Hot stream outlet
	HX5	94.0	3.4	3.4	Hot stream outlet
					
**Case 3**	HX1	66.6	12.5	9.8	Heat exchanger inside
	HX2	51.5	12.9	12.9	Hot stream outlet
	HX3	96.8	14.5	14.5	Hot stream outlet
	HX4	86.3	8.2	8.2	Hot stream outlet
	HX5	92.5	3.6	3.6	Hot stream outlet
					
**Case 4**	HX1	54.1	6.3	−5.9	Infeasible
	HX2	41.4	6.4	6.4	Hot stream outlet
	HX3	82.4	8.1	8.1	Hot stream outlet
	HX4	72.5	1.9	1.9	Hot stream outlet
	HX5	84.8	−2.7	−2.7	Infeasible
					
**Case 5**	HX1	42.1	0.3	−19.8	Infeasible
	HX2	31.2	−0.9	−0.9	Infeasible
	HX3	68.5	0.9	0.9	Hot stream outlet
	HX4	59.2	−5.2	−5.2	Infeasible
	HX5	76.4	−9.8	−9.8	Infeasible

**Fig. 1:**
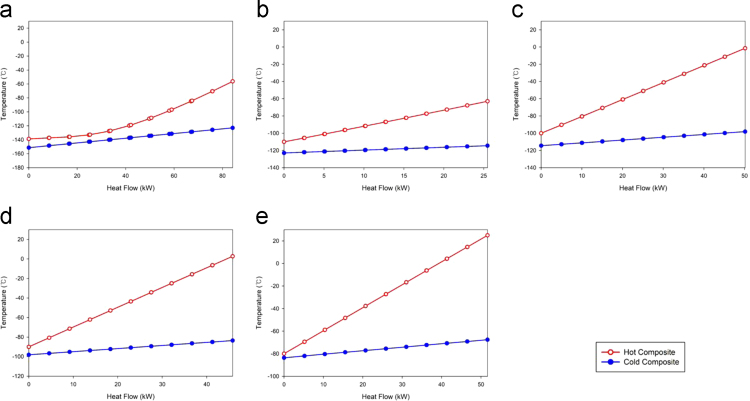
Composite curves for the Case 3: (a) HX1, (b) HX2, (c) HX3, (d) HX4, and (e) HX5.

For the HX2, HX3, HX4, and HX5, heat is transferred from the vapor phase air to the liquid phase LNG. On the other hand, the vapor air is liquefied by the HX1, thus the phase change occurs inside the heat exchanger. The heat exchanging simulation results represent that the pinch points are shown in hot stream outlet of the heat exchanger when the phase does not change. However, the pinch point can be shown not only inlet or outlet but also inside the heat exchanger when the phase changing occurs. It can be well illustrated by the composite curve which is the heat flow to the temperature diagram. The composite curves for heat exchanging are one of the most efficient tools for chemical processes, especially for the heat exchanging dominant processes [2]. The detailed composite curves of the Case 3 are shown in Fig. 1. Note that, the Case 3 is the baseline case of the Ref. [1].

**Fig. 2:**
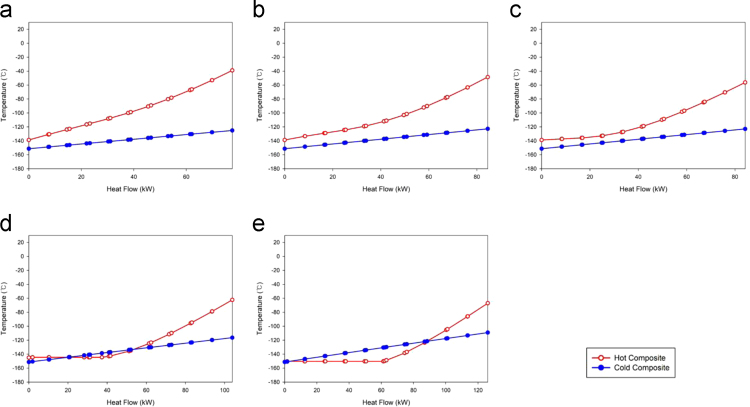
Composite curves for the HX1: (a) Case 1, (b) Case 2, (c) Case 3, (d) Case 4, and (e) Case 5.

Fig. 2 illustrates the heat exchanger with phase changing, HX1, for every case. For the Case 4 and the Case 5, the hot stream temperatures are higher than cold stream temperatures at the inlet and the outlet of the heat exchangers. However, the temperature crosses are occurred inside the heat exchangers.

The temperature crosses are shown inside the heat exchangers for Case 4 and Case 5.
